# Health disparities in outcomes of pediatric systemic lupus erythematosus

**DOI:** 10.3389/fped.2022.879208

**Published:** 2022-10-14

**Authors:** Emily Vara, Mileka Gilbert, Natasha M. Ruth

**Affiliations:** Medical University of South Carolina, Charleston, SC, United States

**Keywords:** health disparities, systemic lupus erythematosus, implementation science, population studies, pediatrics

## Abstract

Healthcare disparities exist throughout the United States, and disparities in healthcare delivery are responsible for a substantial portion of preventable morbidity and mortality. SLE disproportionately affects racial and ethnic minoritized groups, including Blacks, Hispanics, and Asians/Pacific Islanders. Specifically, Black females have a 3 to 4-fold increased risk of developing SLE than White females. Population studies funded through the Centers for Disease Control have examined variations in disease outcomes among the different populations around the United States. For example, studies have shown that lupus nephritis, anti-phospholipid syndrome, and thrombocytopenia are more likely to affect racial and ethnic minorities than Whites. In addition, the Center for Disease Control WONDER (Wide-ranging Online Data for Epidemiologic Research) database found SLE was the seventh leading cause of death for all women aged 15–25 years and the fifth leading cause of death for African American and Hispanic females. From these studies, we know SLE primarily affects racial and ethnic minorities, but we do not know why these groups are at increased risk of developing the disease or have worse outcomes. By examining the underlying mechanisms of health disparities within our patient populations and mitigation strategies, we will further understand and provide better treatment for our patients. This review will discuss current research related to health disparities and health outcomes in childhood-onset SLE (cSLE).

## Introduction

Healthcare disparities exist throughout the United States, and disparities in healthcare delivery are responsible for a substantial portion of preventable morbidity and mortality (1). SLE disproportionately affects racial and ethnic minoritized groups, including Blacks, Hispanics, and Asians/Pacific Islanders. Specifically, Black females have a three to four-fold increased risk of developing SLE than White females (2, 3), Population studies funded through the Centers for Disease Control have examined variations in disease outcomes among the different populations around the United States. For example, studies have shown that lupus nephritis, anti-phospholipid syndrome, and thrombocytopenia are more likely to affect racial and ethnic minorities than Whites (3–5). In addition, the Center for Disease Control WONDER (Wide-ranging Online Data for Epidemiologic Research) database found SLE was the seventh leading cause of death for all women aged 15–25 years and the fifth leading cause of death for Black and Hispanic females. From these studies, we know SLE primarily affects racial and ethnic minorities, but we do not know why these groups are at increased risk of developing the disease or have worse outcomes. By examining the underlying mechanisms of health disparities within our patient populations and mitigation strategies, we will further understand and provide better treatment for our patients. This review will discuss current research related to health disparities in health outcomes of childhood-onset SLE (cSLE) and quality improvement strategies to address disparities.

## Methodology

**Figure 1:**
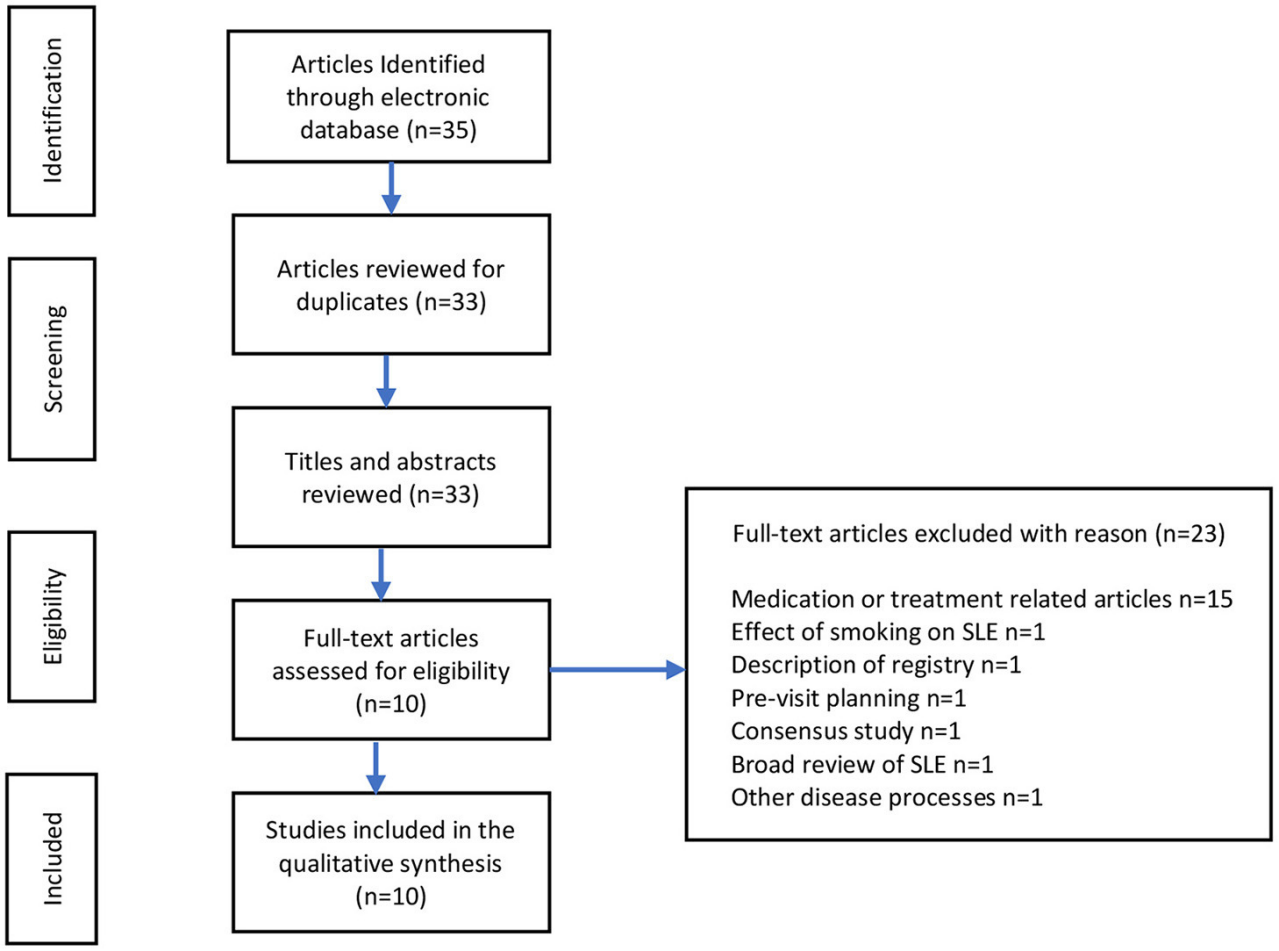
Flow chart for PubMed query for pediatric SLE, health disparities, and patient outcomes.

We performed a PubMed search (Figure 1) with the query including pediatric/children/child, systemic lupus erythematosus/SLE/lupus, health disparities/social vulnerability/adverse childhood events/ACE, and patient outcomes. The search resulted in 33 articles from 2010 to present, of which 10 articles were of relevance. The eligibility of studies focused on pediatric SLE or adult SLE studies that assessed SLE outcomes and disparities. Exclusion criteria included articles discussing medication treatment regimens, including pharmacokinetics and safety and efficacy of new treatment regimens, description of patient registries, and effect of smoking on SLE. There were limited papers focused on cSLE, health disparities, and patient outcomes, therefore we included a few studies pertaining to adults that we felt were relevant. One adult study pertained to the history of pediatric outcomes (adverse childhood events). Quality improvement studies, including a review of a quality framework, were also included due to the relevance in health outcomes and the discussion of three different areas where healthcare quality improvement can be implemented.

## Review of the literature

At this time, there is limited published research investigating disparities in patient outcomes in cSLE. In assessing health disparities in patient outcomes, the primary studies have been large population-based studies, looking at demographics of hospitalizations and associated comorbidities. There is limited data looking specifically at SLE disease activity and health disparities. Another vital area of research in health disparities and outcomes involves improving patient outcomes using care continuum models and quality improvement/ implementation science. Below, we will discuss current health disparities research using population-based studies. We will then transition to studies discussing quality improvement and implementation science to improve patient outcomes.

In assessing health disparity outcomes, we will discuss three studies that examined different patient outcomes. In the first study, Son et al. (6) look at the rate of hospital/ICU admissions, end-stage renal disease, and mortality in SLE. In the second study, Knight and her colleagues discuss health disparities in SLE in relation to depression and anxiety. Lastly, in the third study, an adult SLE study, DeQuattro et al. (7) examines adverse childhood events (ACEs) and their relationship with SLE.

With the assessment of disparities in patient outcomes, Son et al. (6). examined the admissions rate to better understand health disparities in child onset SLE (cSLE). Son et al. investigated whether there was an association between sociodemographic information (Table 1) and variation in the volume of admissions for child onset SLE (6). The outcomes of interest included ICU admissions, ESRD, and in-hospital mortality. The study used pediatric health systems (PHIS) database that included hospitalization information from 43 free-standing pediatric hospitals within the United States. Admission data was analyzed for children between the age of 3 and 18 years with at least 1 International Classification of Diseases (ICD)- 9 code for SLE between January 2006 and September 2011.

**Table 1:** Patient demographics for study evaluating cSLE outcomes from PHIS database from Son et al. (6).

**Demographics**	
**Race**	
White	39%
Black	35.4%
Asian	5.3%
**Ethnicity**	
Hispanic	26.6%
**Insurance**	
Yes	85%
Public insurance	53%

The outcomes of interest in the assessment of sociodemographic health disparities using the PHIS data set, included ICU admissions, ESRD, and in-hospital mortality. There were 10,724 admissions among 2,775 patients in the 43 pediatric hospitals within the United States.

The average follow-up for patients was approximately 1 year. Roughly 85% of patients had insurance, of which 53% of patients had public insurance. A multivariable logistic regression model was performed and controlled for patient gender, age, race, ethnicity, insurance type, hospital volume, US census region, and severity of illness. In the study, Hispanics were noted to have longer length of stays, more readmissions, and increased in-hospital mortality. There was also a significant association between Black patients and increased ICU admissions. Regarding ESRD, about 5.8% of patients with cSLE developed ESRD, with Black and Hispanic patients at higher risk for ESRD. In hospital mortality was 1.5% (*n* = 41), of which 11% of the patients had ESRD. Black patients were also noted to be significantly over-represented in the SLE hospitalizations, accounting for 43.6% of all cSLE admissions. A limitation of the study is we do not know to what degree Black patients were overrepresented in hospitalizations relative to SLE prevalence, or why. There are likely unmeasured risk factors at play, including potentially modifiable social and environmental factors, more so than genetic susceptibility. The Southern region of the United States had a significantly larger population of cSLE patients with renal disease (6). This study by Son et al. (6) demonstrated differences in SLE outcomes based on sociodemographic factors within the United States. Specifically, it noted increased risk for ESRD and mortality in Black and Hispanic minority population groups with SLE. Most recently, a follow-up study evaluated PHIS data from 2006-2019, demonstrated overall improvement in frequency of ESRD and dialysis but continued racial disparities when it comes to ESRD and dialysis and need for interventions to address disparities in renal outcomes (8).

Another examination in health disparities and outcomes in SLE patients, involves the assessment of depression and anxiety. Knight et al., performed a population-based study evaluating the prevalence of depression and anxiety in pediatric patients aged 10 to 18 years old with SLE (9). Data was obtained from the US Medicaid Analytic Extract database from 2006 to 2007. The study included 970 children with SLE, of which 15% identified White, 42% identified Black, 27% identified Latino, and 16% identified as other races or ethnicities (which included Asian, Pacific Islander, Native American, and other). The study used ICD-9 codes for depression, anxiety, adjustment disorder, and other psychiatric disorders to determine the prevalence of mental health disorders in cSLE.

Of the 970 patients, 19% had depression, 7% had anxiety, 6% had acute adjustment disorder, and 18% had other psychiatric disorders. The analysis was adjusted for age, sex, urban vs. rural environment, presence of lupus nephritis, presence of seizure/strokes, glucocorticoid use, and the number of outpatient visits. In the adjusted analysis, there was a significant difference in diagnosis of depression and anxiety in Black patients compared to White patients, with Black patients less likely to be diagnosed with depression and anxiety. There was no significant difference between Whites and other races or ethnicities (9).

Among the 970 patients, 20% were prescribed antidepressants, 7% were prescribed anxiolytics, and 6% were prescribed antipsychotics. For the patients diagnosed with a psychiatric diagnosis, approximately 61% were prescribed at least 1 psychotropic medication. For the patients who did not have a psychiatric diagnosis, approximately 17% had been prescribed at least 1 psychotropic medication, leading to concern for under-diagnosis vs. other medical indications. Additionally, when prescription demographics were further classified, Black patients were significantly less likely to be prescribed anxiolytics. The study also noted a significant increase in depression and anxiety in patients with increased medical visits (9).

Overall, the study illustrates the high burden of depression (19%) and anxiety (7%) in cSLE patients, which was significantly more than the overall prevalence of depression and anxiety noted in the general Medicaid population (9). The study also illustrates the concern for decreased treatment of depression and anxiety in Black patients within the Medicaid cohort. A study in adolescent psychiatry also noted racial and ethnic minorities were less likely to be prescribed treatment for depression (10). There could be numerous reasons for treatment, such as the use of therapy rather than medication. Additionally, family or culture stigma related to mental health and treatment may also play a role in treatment of depression (9, 10). Within the clinical setting, language concordance between patient and provider could also be a factor (10).

An additional area of health disparities research is whether adverse childhood events (ACEs) contribute to the development of SLE. Most recently, DeQuattro et al. examined whether there was an association between ACEs and systemic lupus erythematosus in adult patients (7). Although this study does not assess the association of ACEs in a pediatric population with SLE, the study was included because of the relevance in the implication of ACEs in association with SLE. The prevalence of ACEs in childhood of patients with SLE from the California Lupus Epidemiology Study (CLUES) was compared to population survey estimates from California's Behavioral Risk Factor Surveillance System (BRFSS). The BRFSS survey at the time of the study did not include neglect; therefore, the neglect portion of the ACE questionnaire was omitted. They then evaluated for associations in overall ACE level with patient-reported outcomes and physician assessed health status measures. A multivariable regression analysis was performed and controlled for sociodemographic, nephritis, and cSLE (7). Results of the study found that the distribution of ACE levels was similar for the CLUES and BRFSS groups (*p* = 0.42). Two potential reasons for the similar prevalence values include selection and survival bias in the CLUES group, with patients with more severe disease activity or lower socioeconomic level less likely to participate in the study (7).

Further examination of the CLUES group and association with SLE disease activity showed the median ACE level was 1 and about 20% of participants had ≥4 ACEs. An ACE level ≥1 was more likely to be seen in an older patient of Hispanic ethnicity or Black race and with a history of lupus nephritis. A patient with an ACE score greater than 0 was also less likely to have a 4-year college degree. It was also noted that patients who reported increased SLE disease activity, damage, and depression had increased ACE levels. There was also a decrease in physical function and health status with increasing ACE levels. Overall, there was a noted “dose-response” association between patient-reported health outcomes and ACE level (7). The associations remained significant even after adjustment for age, sex, race and ethnicity, cSLE, and educational attainment. However, interestingly there was not a significant association between ACE levels and SLE disease activity with physician associated measures. It is unclear why there was an association between patient reported outcomes and ACE level and not physician reported outcomes. It was speculated overall health status and mood may also play a role in the patient reported outcomes and impact the score (7). This study is one of the first to evaluate ACE levels in SLE patients and demonstrated overall higher ACE levels with patient reported outcomes. ACE levels are an important consideration for future research because of the association between toxic stress and worse long-term health outcomes, particularly in people with chronic diseases.

An additional area of research in health disparities involves ways to improve patient outcomes. The next two studies focus on using frameworks to improve outcomes for SLE patients. A key part to the studies is examining how improving health disparities can improve patient outcomes.

For the first study, Bartels et al. (11) were interested in evaluating retention in care as a mechanism to improve patient outcomes. The retention in care model was a framework for human immunodeficiency virus (HIV) from World Health Organization (WHO), Institute of Medicine (IOM), and Centers for Disease Control and Prevention (CDC) to improve patient outcomes. By developing strategies related to information gained from the continuum of care model, viral suppression has improved from 61 to 80% between 2012 and 2016. Bartels et al. (11) were interested in the HIV continuum of care model because HIV disproportionately impacts young adults in minority groups and socioeconomically disadvantaged. They wanted to develop and validate a similar model for SLE. They were interested in the relationship of race, socioeconomic disadvantage, and retention in care. The 5 steps of the SLE care continuum include diagnosis of SLE, link to rheumatology care, retain in care, immunotherapy, and low disease activity/damage (11).

In developing the SLE care continuum model, the primary care clinic visit was of importance to assess for other comorbidities. Patient demographics were notable for 91% female, 39% Black, 5% other, and 4% Hispanic. Black patients were more likely to live in disadvantaged neighborhoods (51 vs. 5% of White patients) and more likely to receive public insurance (75 vs. 40% for White patients). In defining retention in care for SLE, they focused on 2 annual visits and 2 lab defined every 6 months. When assessing predictors for the care model, living in a disadvantaged neighborhood was the strongest predictor in lower retention (OR 0.41, 95% CI 0.18, 0.93) as opposed to an individual's race. In general, the neighborhood has a significant impact on one's health, including access to food, pharmacies, clinics, as well as safety in a patient's daily life. Being aware that neighborhoods play an important factor for retention in care, knowing which neighborhoods have limited resources is an important consideration. The Centers for Disease Control and Prevention (CDC) created the social vulnerability index (SVI), which compiles 15 social factors, based on census tract information, into an interactive map to identify communities that may need more support. Using the SVI could be a helpful screening tool for clinicians to determine which patients are at increased risk for lower retention in care and develop strategies in clinic to mitigate effects and improve outcomes (12). Overall, authors concluded that success reducing disparities in HIV highlights the need to measure SLE retention in care as a necessary first step toward designing interventions, policies, and building patient partnerships to eliminate lupus outcome disparities (11).

A review from Lawson used Donabeidan's framework for assessment of healthcare quality in SLE patients (13). The Donabeidan framework divides factors into 3 areas: structures, processes, and outcomes. Using this framework allows further understanding of potential factors and underlying mechanism impacting SLE outcomes and develop strategies for improvement. Structure includes attributes related to the clinical setting, such as facilities, financial resources, and equipment. Accessing care, such as making to clinical appointments with a rheumatologist, was associated with age, insurance status, socioeconomic status, distance to healthcare providers, and neighborhood factors. A population study using United States Renal Data System (USRDS) data in California found that the incidence of ESRD from SLE varied by zip code (14). There was an increased incidence of ESRD in zip codes with a greater proportion of public insurance or uninsured and a higher incidence of hospitalizations for conditions treated in ambulatory clinics. These findings were independent of an individual person's socioeconomic status. Additionally, geographic distance to a medical center can contribute to decrease in care. In the Lupus outcomes study from 2002-2004, Medicaid patients often traveled further to their rheumatologist than those with private insurance. The patients were also more likely to obtain care for their SLE through their primary care practitioner or the Emergency Department. An example of using a factor within the structure branch of the framework is developing a coordinated clinic appointments for patients with multiple subspecialists. With coordinated appointments, they have shown the potential of reducing hospitalizations, cost, and improve quality in chronic illnesses (13).

The second factor included in the Donabeidan framework is process of care. Process of care includes actions performed in giving and receiving care. The majority of quality improvement projects focus on process of care because actionable targets can be easily created. The development of cSLE quality indicators is an example of process care actions. The quality indicators for cSLE include 26 indicators focused on diagnosis, health maintenance measures, diagnosis and treatment for lupus nephritis, general preventive strategies, surveillance for medication safety, counseling and evaluation of cardiovascular risk, and transition planning. Since the development of the 26 quality indicators, there have been a couple of studies that have investigated adherence with specific quality indicators (15). Authors also remarked that even though quality indicators have emerged as an important tool to measure quality, further validation is necessary to define not only their validity but also feasibility in clinical practice and whether they are associated with improved clinical outcomes (13).

In a benchmarking study evaluating the adherence of quality indicators for cSLE, 7 centers from the United States, India, and Brazil, Mina et al. (16) found wide variability in assessing cardiovascular risks factors (21–100%), bone mineral density evaluation (7–90%), and discussion of sun exposure prevention (58–99%) among the different centers. Within the United States, there was not a significant difference among the 4 centers between private and public insured patients in quality indicators (16). In addition, Harris et al. (17) evaluated the cSLE quality indicators: hydroxychloroquine use, vitamin D recommendations, meningococcal vaccination, pneumococcal vaccination, influenza vaccination, ophthalmology screening, and bone mineral density evaluation at a single center. Adherence to quality indicators ranged between 28.6 and 94.4%, with bone mineral density evaluation having the lowest adherence and Plaquenil recommendations having the highest adherence. During this time, they also compared the quality indicators to SLE disease damage and race. There was not a significant association between the individual quality indicators and race or ethnicity, but there was a significant association between disease damage and minority race and ethnicity. The study was at a single center and may have had inadequate power to evaluate whether following quality indicators leads to improvement in racial health disparities. Further studies at other centers will be crucial in monitoring whether quality indicators improve racial health disparities and whether additional quality indicators or barriers improve health disparities (17).

The pneumococcal vaccine rate is one of the quality indicators often with poor adherence. At Nationwide Children's hospital, Sivaraman et al. (18) used quality improvement methodology to improve pneumococcal vaccine rate amongst cSLE patients. They determined the barriers (updated electronic health record with vaccine records and provider understanding of vaccine administration recommendations) for administering pneumococcal vaccine, and within 18 months their administration rate improved from 2.5 to 87% (18). The study highlights evaluating adherence to quality indicators, assessing for barriers to improved health outcomes, and how to effect clinical change to provide a sustained improvement in patient health.

## Future directions

SLE is a chronic disease that disproportionately affects racial and ethnic minority groups. Current research evaluating health outcomes in cSLE is limited but does show that minoritized populations are disproportionately affected and have worse outcomes, such as increased frequency of hospitalizations, ESRD, and mortality in Hispanic and Black patients (6). Additionally, SLE patients are at increased risk for depression and anxiety, which can impact medication adherence.

The science of health care disparities is unique. To be done well, it needs to include a comprehensive investigation into an individual's circumstances and the communities we serve and our health care systems over time. Recognition that health disparities are driven by social and economic inequities that are embedded into our health care system is of utmost importance.

Currently, there is limited research examining pediatric health disparities in childhood onset SLE and outcomes. Future research is needed to evaluate the association of health disparities and cSLE disease outcomes. It is important to evaluate and determine solutions to pediatric health disparities from different angles. For example, multi-center observational studies need to be performed to evaluate the association between SLE disease outcomes and pediatric health disparities. Additionally, the use of a standardized tool, such as children's healthcare of Philadelphia's childhood lupus index, to assess care quality for cSLE will allow universal data collection across centers to better assess cSLE outcomes (19). It will allow for a more comprehensive evaluation of quality indicators and whether or not they are indeed an important tool to measure quality and moreover, if they are even associated with improved outcomes. From there we can see what areas specific for the medical center and nationally need improvement.

One area of patient outcomes noted in previous studies is that larger medical centers often have improved patient outcomes compared to smaller centers (19). Further investigation examining why larger centers have improved outcomes is an important area to pursue, such as examining whether specific resources improve outcomes. Often, smaller centers do not have access to a social work or a lupus patient navigator, which play a critical role in assessing for health disparities and their knowledge in regional resources. An intervention to consider would be funding opportunities for small centers to provide a social worker or lupus patient navigator and see if cSLE outcomes improve. Once we understand cSLE outcomes using a standardized tool, we can evaluate areas needing improvement, monitor for areas of disparities, and use implementation science and quality improvement frameworks to improve patient outcomes.

As a mechanism to improve health disparities, implementation science and quality improvement have been used to improve health outcomes (11, 13). Implementation science and quality improvement frameworks provide flexibility and the ability to localize to regional issues related to health disparities. As part of the design, process, and implementation phases, it is important to determine the best approach and modifications, if necessary, that are inclusive and reflective of the unique aspects of health disparity populations in the real-world setting (1, 20). Over the next few years, further research will be available that will provide more insight into interventions to lessen the effect of health disparities and better advocate for our patients regarding health policy.

## Author contributions

All authors listed have made a substantial, direct, and intellectual contribution to the work and approved it for publication.

## Funding

This study received a training grant through NIH/NIAMS T32 AR050958.

## Conflict of interest

The authors declare that the research was conducted in the absence of any commercial or financial relationships that could be construed as a potential conflict of interest. The reviewer JC declared a shared committee with the author MG to the handling editor.

## Publisher's note

All claims expressed in this article are solely those of the authors and do not necessarily represent those of their affiliated organizations, or those of the publisher, the editors and the reviewers. Any product that may be evaluated in this article, or claim that may be made by its manufacturer, is not guaranteed or endorsed by the publisher.
